# COVID-19 infection: epidemiological, clinical, and radiological expression among adult population

**DOI:** 10.1186/s43055-020-00341-9

**Published:** 2020-11-25

**Authors:** Eman Ragab, Asrar Helal Mahrous, Ghadeer Maher El Sheikh

**Affiliations:** 1 Department of Radiodiagnosis, Faculty of Medicine, Menoufia University, Shibin el Kom, Menoufia 32511 Egypt; 2 Department of Chest Diseases and Tuberculosis, Faculty of Medicine, Menoufia University, Shibin el Kom, Egypt; 3 Department of Public Health and Community Medicine, Faculty of Medicine, Menoufia University, Shibin el Kom, Egypt

**Keywords:** COVID-19, Computed tomography, Pneumonia, Coronavirus infection

## Abstract

**Background:**

High-resolution computed tomography (HRCT) has proved to be an important diagnostic tool throughout the COVID-19 pandemic outbreaks. Increasing number of the infected personnel and shortage of real-time transcriptase polymerase chain reaction (RT-PCR) as well as its lower sensitivity made the CT a backbone in diagnosis, assessment of severity, and follow-up of the cases.

**Results:**

Two hundred forty patients were evaluated retrospectively for clinical, laboratory, and radiological expression in COVID-19 infection. One hundred eighty-six non-severe cases with home isolation and outpatient treatment and 54 severe cases needed hospitalization and oxygen support. Significant difference between both groups was encountered regarding the age, male gender, > 38° fever, dyspnea, chest pain, hypertension, ≤ 93 oxygen saturation, intensive care unit (ICU) admission, elevated D-dimer, high serum ferritin and troponin levels, and high CT-severity score (CT-SS) of the severe group. CT-SS showed a negative correlation with O_2_ saturation and patients’ outcome (*r* − 0.73/*p* 0.001 and *r* − 0.56/*p* 0.001, respectively). Bilateral peripherally distributed ground glass opacities (GGOs) were the commonest imaging feature similar to the literature.

**Conclusion:**

Older age, male gender, smoking, hypertension, low O_2_ saturation, increased CT score, high serum ferritin, and high D-dimer level are the most significant risk factors for severe COVID-19 pneumonia. Follow-up of the recovered severe cases is recommended to depict possible post COVID-19 lung fibrosis.

## Background

Coronavirus family is enveloped positive-stranded RNA viruses accustomed to cause usual non-severe upper respiratory tract symptoms, similar to common cold infection. Unfortunately, new versions that have emerged from animal reservoirs over the last two decades led to serious and widespread morbidities and mortalities [1].

By the end of 2019, a novel coronavirus was recognized to result in an illness outbreak that emerged in China. The virus is currently known as the severe acute respiratory syndrome coronavirus 2 (SARS-CoV-2). The resultant disease is called coronavirus disease 2019 (COVID-19). In March 2020, the World Health Organization (WHO) declared the COVID-19 outbreak as a pandemic [2, 3].

The host receptor for SARS-CoV-2 cell entry is the same as for SARS-CoV, the angiotensin-converting enzyme 2 (ACE2) [4]. Signs and symptoms of coronavirus disease 2019 (COVID-19) may manifest 2 to 14 days following exposure. Common presentation can include fever, cough, and tiredness. Early symptoms of COVID-19 may be just loss of taste or smell. Other symptoms like shortness of breath or difficulty breathing, muscle ache, chills, sore throat runny nose, headache, or chest pain have been reported [5].

Many articles have been published as a descriptive demonstration for imaging findings in cases with COVID-19 infection with emphasis on computed tomography (CT) in screening, diagnosis, and follow-up of COVID-19 pneumonia [6–9]. High-resolution computed tomography (HRCT) is considered a backbone tool in management of the disease [10]. The purpose of the current study was to correlate the clinical expression of the disease and the HRCT findings among adults.

## Methods

### Cases

This retrospective study included 240 participants from 25 March 2020 till 5 July 2020. One hundred forty were males (58.3%) and 100 were females (41.7%). Age range was 18–85 years with mean ± SD = 45.82 ± 16.74. Clinical, laboratory, and radiological evaluation occurred within the first 5 days of the onset of the symptoms. The Institutional Research Ethics committee has approved the study, and a written informed consent was obtained from each patient.

### Epidemiological and clinical points of view

The patients were included according to the following: (1) epidemiological background, traveling to areas with case reports or exposure to a confirmed case within 14 days prior to the onset of symptoms; (2) clinical and laboratory manifestations, the patients underwent complete history taking, general and local examination, laboratory tests including complete blood count (CBC), C-reactive protein (CRP), serum ferritin, D-dimer, and liver and kidney function tests; (3) imaging criteria for COVID-19 pneumonia; (4) real-time transcriptase polymerase chain reaction (RT-PCR) of the nasopharyngeal swab for 188 cases.

### Image acquisition and analysis

HRCT of the chest was done using a 160 slice Multi-Detector Toshiba CT Aquilion Prime scanner. Patients were in supine position with head first. Image acquisition parameters were 100 kV, 250 mAs, FOV = 400 × 460 mm, slice thickness 1.0 mm, and inter-slice gap 1.0 mm. The images were reviewed on VITREA workstation for interpretation.

**Fig. 1:**
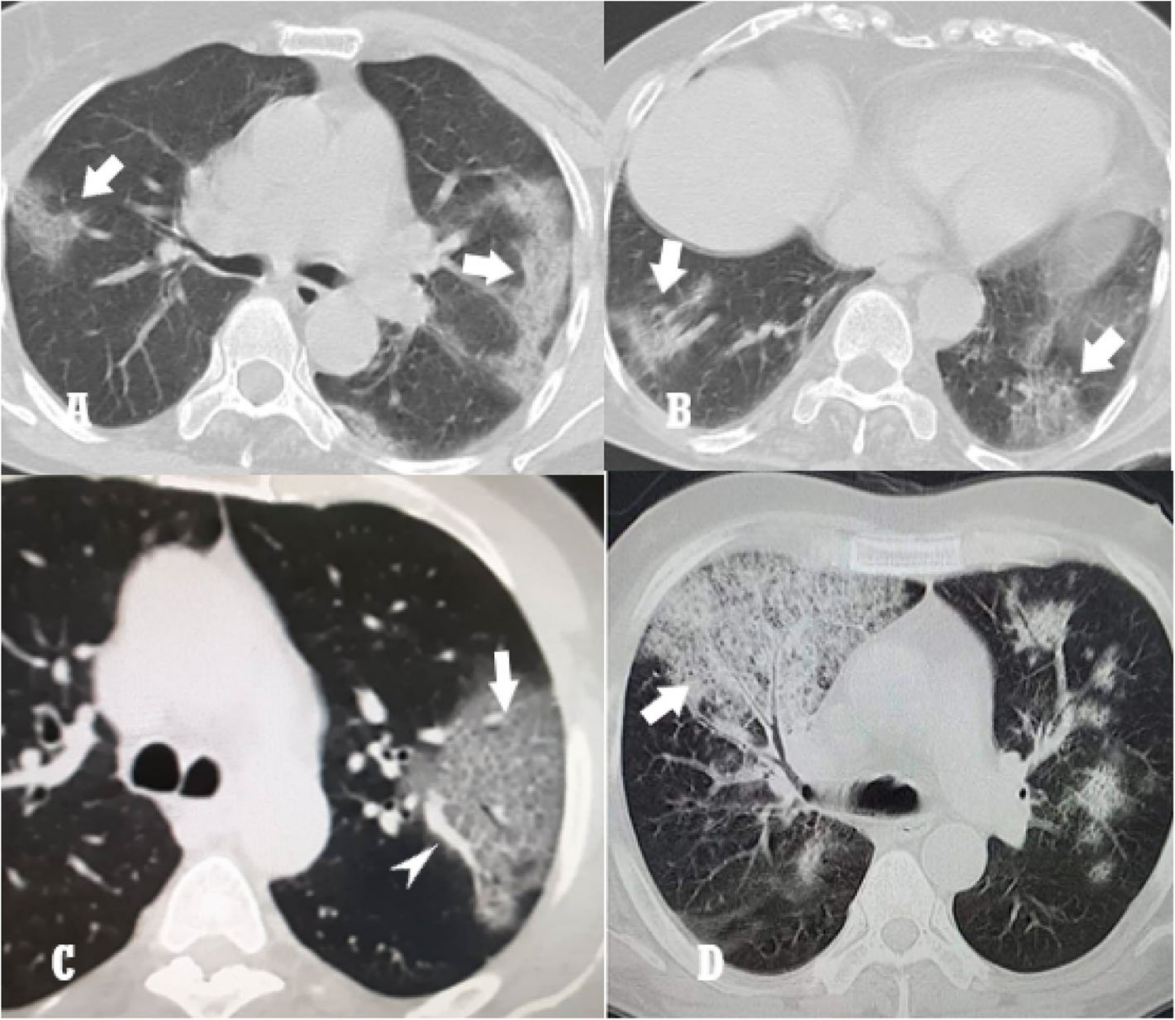
**a** and **b** Ground glass opacities. **c** and **d** Crazy paving pattern. Note the vascular prominence (arrow head in **c**)

**Fig. 2:**
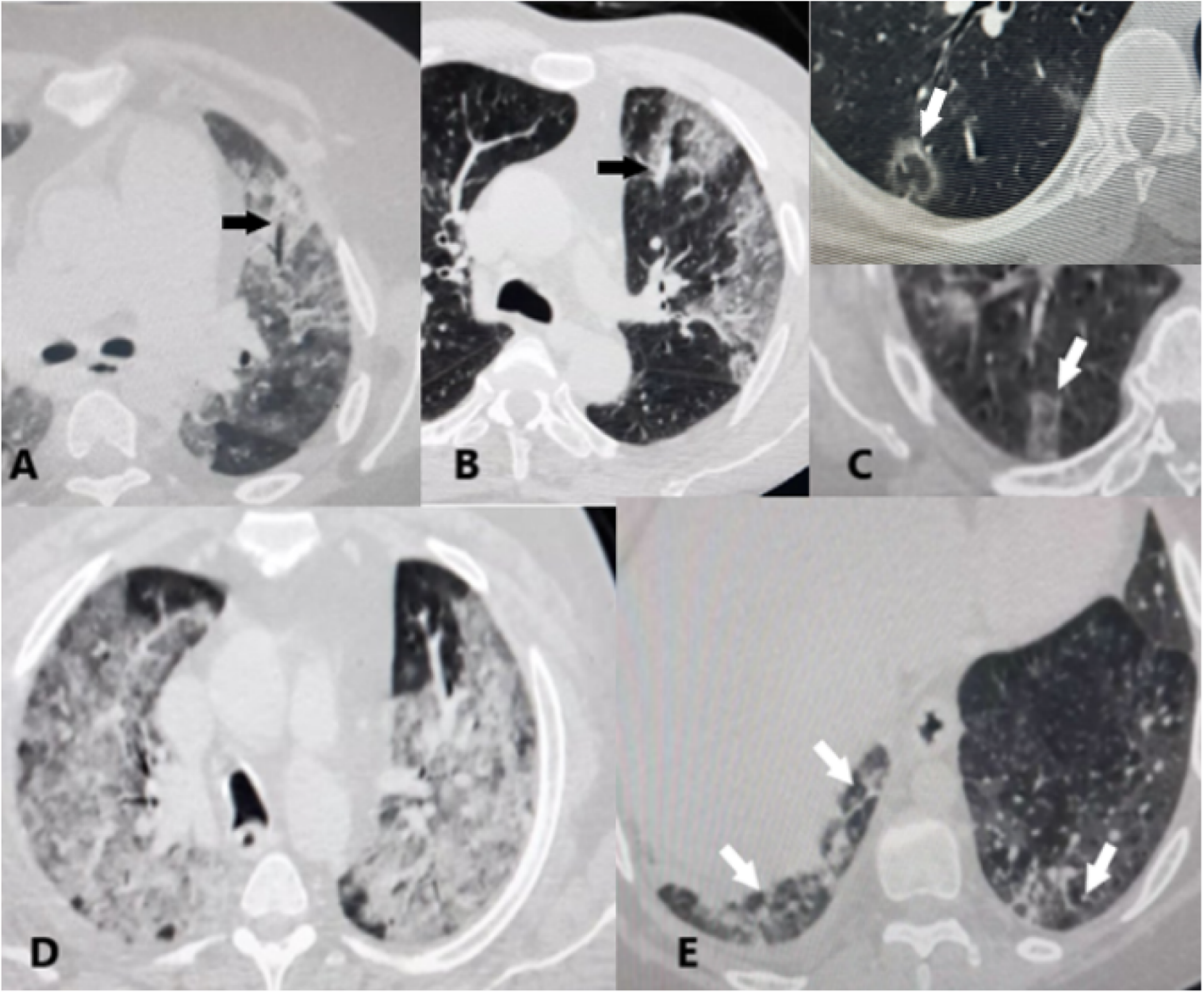
**a** Air bronchogram sign. **b** Vascular prominence. **c** Reverse halo sign. **d** White lung sign. **e** Septal lines

Each chest scan was assessed for laterality involvement (unilateral/bilateral), number of lung lobes affected (unilobar/bilobar/multilobar), distribution/location of the lesions (peripheral = outer third/central = inner two thirds), lesion patterns (ground glass opacities/consolidations/crazy paving/septal lines), special signs (air bronchogram/vascular prominence/white lung/reverse halo), and other imaging findings (pleural effusion/mediastinal lymphadenopathy) (Figs. 1 and 2).

Determination of the CT severity score (CT-SS) is as follows: by anatomy, both lungs consist of five lobes. Each lobe was assessed for affection, score 0 = 0% involvement; score 1 if < 5% involvement; score 2 if 5% to < 25% involvement; score 3 if 25 to < 50% involvement; score 4 if 50 to < 75% involvement; and score 5 if ≥ 75% involvement. Then, calculation of the total score that ranged from 0 to 25 [11, 12].

### Follow-up CT

**Fig. 3:**
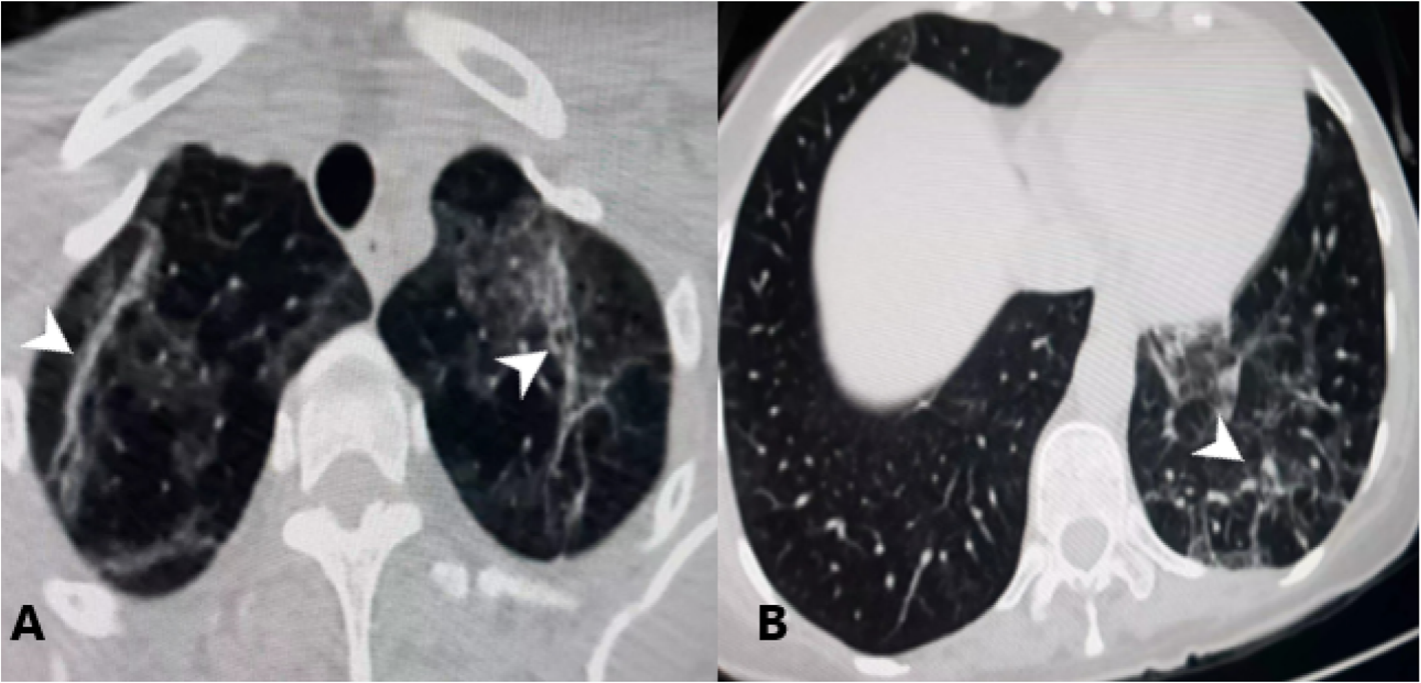
Strands of fibrosis (arrow heads)

Eighteen cases underwent follow-up after discharge (about 5 weeks from the onset of the illness) due to recurrent dyspnea, and their CT showed strands of fibrosis as in Fig. 3.

### Statistical analysis

The descriptive statistics are mean, standard deviation, and median for continuous data. The statistics for categorical variables are counts and percentages. Student’s *t* test and Mann-Whitney *U* test were performed for continuous variables, and the χ^2^ test and Fisher exact test were used for categorical variables. *Z* test was used to compare two sample proportions. Spearman’s test was used to analyze the correlation between measurement data and ordinal variables while the Pearson correlation was used to evaluate the linear relationship between two continuous variables.

Multivariable binary logistic regression analyses were used to assess the association between age, gender, smoking history, O_2_ saturation, CT score, CBC, serum ferritin and D-dimer level, and the dependent variable of severity of disease. The odds ratio (OR) along with the 95% CI were reported. Odds ratio (OR) is a measure of association between exposure and an outcome. The OR represents the odds that an outcome will occur given a particular exposure, compared to the odds of the outcome occurring in the absence of that exposure. A *P* value of less than .05 was regarded as statistically significant. All statistical analyses were performed using SPSS 20.0 for Windows (SPSS, Inc, Chicago, IL).

## Results

A total of 240 cases diagnosed as COVID-19 infection (188 underwent RT-PCR and 113 cases were positive representing 60.1%) were included. The patients were divided into two subgroups severe and non-severe cases whether the case needed ventilatory oxygen support at time of presentation. This was according to the 2019 clinical practice guideline from the Infectious Diseases Society of America and the American Thoracic Society for diagnosis and treatment of adults with community-acquired pneumonia [13].

One hundred eighty-six non-severe cases were recorded; their mean age ± SD = 41.88 ± 14.58 years, including 96 males (51.6%) and 90 females (48.4%). The severe cases counted 54 with mean age ± SD = 59.37 ± 16.83 years, 44 males (81.5%) and 10 females (18.5%). There was significant difference with higher age and large male percentage among the severe group [*p* < 0.0001 and 0.006 respectively]. The severe cases were admitted for 10–21 days, and 28 cases died.

In the analysis of the symptoms that belong to both groups, loss of taste and smell, sore throat, and runny nose were significantly common in non-severe cases (*p* < 0.0001 for all). On the other hand, dyspnea, chest pain, and expectoration were significantly common among severe cases (*p* < 0.0001, 0.008, and < 0.0001 respectively). Regarding the associated co-morbidities, systemic hypertension and cerebrovascular compromise were significantly encountered among severe cases (*p* 0.002 and 0.008 respectively) (Table 1).

**Table 1:** Demographic and clinical characteristics of the patients

	All patients (***n*** = 240)	Non-severe cases (***n*** = 186)	Severe cases (***n*** = 54)	***P*** value
**Age (years)**				.000
Mean ± SD (median)	45.82 ± 16.74 (44.0)	41.88 ± 14.55 (42.00)	59.37 ± 16.67 (62.00)	
Range	18–85	18–75	20–85	
**Age groups**				.000
≥ 65 years	36 (15.0%)	10 (5.4%)	26 (48.1%)	
< 65 years	204 (85.0%)	176 (94.6)	28 (51.9%)	
**Gender**				.000
Male	140 (58.3%)	96 (51.6%)	44 (81.5%)	
Females	100 (41.7%)	90 (48.4%)	10 (18.5%)	
**Smoking history**				.006
Smoker	116 (48.3%)	81 (43.5 %)	35 (64.8%)	
Non-smoker	124 (51.7%)	105 (56.5%)	19 (35.2%)	
**Source of infection**				0.67
Known source	148 (61.7%)	116 (62.4 %)	32 (59.3%)	
Unknown source	92 (38.3 %)	70 (37.6 %)	22 (40.7%)	
**Body temperature**				.001
≤ 38 °C	142 (59.2%)	122 (65.6%)	20 (37.0%)	
> 38–39 °C	94 (39.2%)	62 (33.3%)	32 (59.3%)	
> 39 °C	4 (1.7%)	2 (1.1%)	2 (3.7%)	
**Malaise**	150 (62.5%)	120 (64.5%)	30 (55.6%)	0.23
**Bone ache/myalgia**	142 (59.2%)	104 (55.9%)	38 (70.4%)	0.06
**Loss of taste/smell**	94 (39.2%)	92 (49.5%)	2 (3.7%)	.000
**Headache**	76 (31.7%)	68 (36.6%)	8 (14.8%)	0.002
**Sore throat**	102 (42.5%)	96 (51.6%)	6 (11.1%)	.000
**Runny nose**	86 (35.8%)	86 (46.2%)	0 (0.0%)	.000
**Dry cough**	202 (84.2%)	164 (88.2%)	38 (70.4%)	0.002
**Dyspnea**	106 (44.2%)	58 (31.2%)	48 (88.9%)	.000
**Chest tightness**	176 (73.3%)	130 (69.9%)	46 (85.2%)	0.02
**Chest pain**	4 (1.7%)	0 (0.0%)	4 (7.4%)	.000
**Expectoration**	38 (15.8%)	16 (8.6%)	22 (40.7%)	.000
**Abdominal pain**	86 (35.8%)	74 (39.8%)	12 (22.2%)	.01
**Diarrhea**	70 (29.2%) 170 (70.8%)	62 (33.3%) 124 (66.7%)	8 (14.8%) 46 (85.2%)	.008
**Cardiovascular diseases**	6 (2.5%)	4 (2.2%)	2 (3.7%)	.52
**Cerebrovascular disorders**	4 (1.7%)	0 (0.0%)	4 (7.4%)	.000
**HTN**	54 (22.5%)	30 (16.1%)	24 (44.4%)	.000
**DM**	92 (38.3%)	66 (35.5%)	26 (48.1%)	.09
**Other diseases**	32 (13.3%)	30 (16.1%)	2 (3.7%)	.01
**O**_**2**_ **saturation**				.000
Mean ± SD	92.43 ± 8.48	96.01 ± 3.44	80.07 ± 9.11	
≤ 93	82 (34.2%)	32 (17.2%)	50 (92.6%)	
> 93	158 (65.8%)	154 (82.8%)	4 (7.4%)	
**ICU admission**	48 (20.0%)	8 (4.3%)	40 (74.1%)	.000
**Disease outcome**				.000
Improvement	212 (88.3%)	186 (100%)	26 (48.1%)	
Death	28 (11.7%)	0 (0.0%)	28 (51.9%)	

*P* values comparing non-severe cases and severe cases are from χ^2^ test, Fisher’s exact test, or Mann-Whitney *U* test. A *P* value of less than 0.05 was regarded as statistically significant

The mean oxygen saturation ± SD was 96.01 ± 3.44 for non-severe cases and 80.07 ± 9.11 for severe cases (*p* < 0.0001). One hundred fifty-eight cases (65.8%) had > 93%, and 82 cases (34.2%) had ≤ 93%. The severe cases showed significantly lower levels (*p* < 0.0001).

According to the laboratory results, serum ferritin, D-dimer, and troponin levels were significantly higher in severe patients compared to the non-severe ones (*p* 0.002, 0.001, and < 0.0001 respectively) (Table 2).

**Table 2:** Laboratory findings of the patients

	All patients (***n*** = 240)	Non severe cases (***n*** = 186)	Severe cases (***n*** = 54)	***P*** value
**Lymphopenia in CBC**	224 (93.3%)	176 (94.6%)	48 (88.9%)	0.13
**High S.ferritin**	144 (60%)	98 (52.7%)	46 (85.2%)	.000
**High D.dimer**	130 (54.2%)	86 (46.2%)	44 (81.5%)	**.**000
**Positive CRP**	238 (99.2%)	186 (100%)	52 (96.3%)	.008
**Elevated LFT/KFT**	8 (3.3%)	4 (2.2%)	4 (7.4%)	.05
**High troponin**	22 (9.2%)	6 (3.2%)	16 (29.6%)	.000
**RT-PCR (done for 188 cases only)**				
	(188)	(142)	(46)	.01
**Positive**	113 (60.1%)	78 (54.9%)	35 (76.1%)	
**Negative**	75 (39.9%)	64 (45.1%)	11 (23.9%)	

*p* values comparing non-severe cases and severe cases are from χ^2^ test or Fisher’s exact test. A *P* value of less than 0.05 was regarded as statistically significant

Regarding CT findings, involvement of both lungs has been reported in 188 cases (78.3%), 48 cases (20%) showed unilateral lung affection, and 4 cases (1.7%) elicited normal lungs. According to lesion location either peripheral involving the outer third or central involving the inner two thirds, 140 cases (58.3%) showed peripheral lung lesions, 8 cases (3.3%) with central lesions, and 88 cases (36.7%) presented peripheral and central lesions as well.

The commonest pattern encountered on CT was the ground glass opacities (GGOs) representing 96.7%, followed by consolidations with 39.2%. Air bronchogram was the commonest special CT sign recorded in 92 cases (38.3%), and the least sign was reverse halo sign in 18 cases (7.5%) (Table 3).

**Table 3:** CT findings of the patients

	All patients (240)	Non severe cases (***n*** = 186)	Severe cases (***n*** = 54)	***P*** value
**Lesion laterality**				0.001
Unilateral lesions	48 (20%)	46 (95.8%)	2 (4.2%)	
Bilateral lesions	188 (78.3%)	136 (72.3%)	52 (27.7%)	
Free CT	4 (1.7%)	4 (100%)	0 (0%)	
**Lesion location**				< 0.000
Peripheral	140 (58.3%)	124 (88.6%)	16 (11.4%)	
Central	8 (3.3%)	8 (100.0%)	0 (0%)	
Both	88 (36.7%)	50 (56.8%)	38 (43.2%)	
**Lesion distribution**				< 0.000
Unilobar	32 (13.3%)	32 (100%)	0 (0%)	
Bilobar	104 (43.3%)	102 (98.1%)	2 (1.9%)	
Multilobar	100 (41.7%)	48 (48.0%)	52 (52.0%)	
**Lesion pattern**				
GGO	232 (96.7%)	180 (77.6%)	52 (22.4%)	< 0.000*
Consolidation	94 (39.2%)	46 (48.9%)	48 (51.1%)	0.83*
Septal lines	44 (18.3%)	36 (81.8%)	8 (18.2%)	< 0.000*
Crazy paving	62 (25.8%5)	54 (87.1%)	8 (12.9%)	< 0.000*
**Special signs**				
Air bronchogram	92 (38.3%)	44 (47.8%)	48 (52.2%)	0.67*
Reverse halo	18 (7.5%)	16 (88.9%)	2 (11.1%)	0.009*
Vascular prominence	28 (11.7%)	14 (50.0%)	14 (50.0%)	1.00*
White lung	24 (10.0%)	0 (0%)	24 (100.0%)	----------
**Other findings**				
Lymphadenopathy	36 (15.0%)	22 (61.1%)	14 (38.9%)	0.19*
Pleural effusion	2 (0.8%)	0 (0%)	2 (100.0%)	----------
**CT score**				
Mean ± SD	12.22 ± 6.02	9.56 ± 3.42	21.41 ± 3.34	< 0.000^#^
Mild (0–7)	68 (28.3%)			
Moderate (8–15)	118 (49.2%)			
Severe (16–25)	54 (22.5)			

*P* values comparing non-severe cases and severe cases are from χ^2^ test or Fisher exact test

*P values comparing non-severe cases and severe cases are from *Z* test

^#^*P* values comparing non-severe cases and severe cases are from t test. A *p* value of less than 0.05 was regarded as statistically significant

Regarding the imaging findings, the non-severe cases showed significant frequency of unilateral pulmonary affection, central location of the lesions, unilobar and bilobar affection, ground glass opacities and septal lines as well as the crazy paving patterns, reverse halo as a special imaging sign, and lower CT severity scores as illustrated in Table 3.

**Fig. 4:**
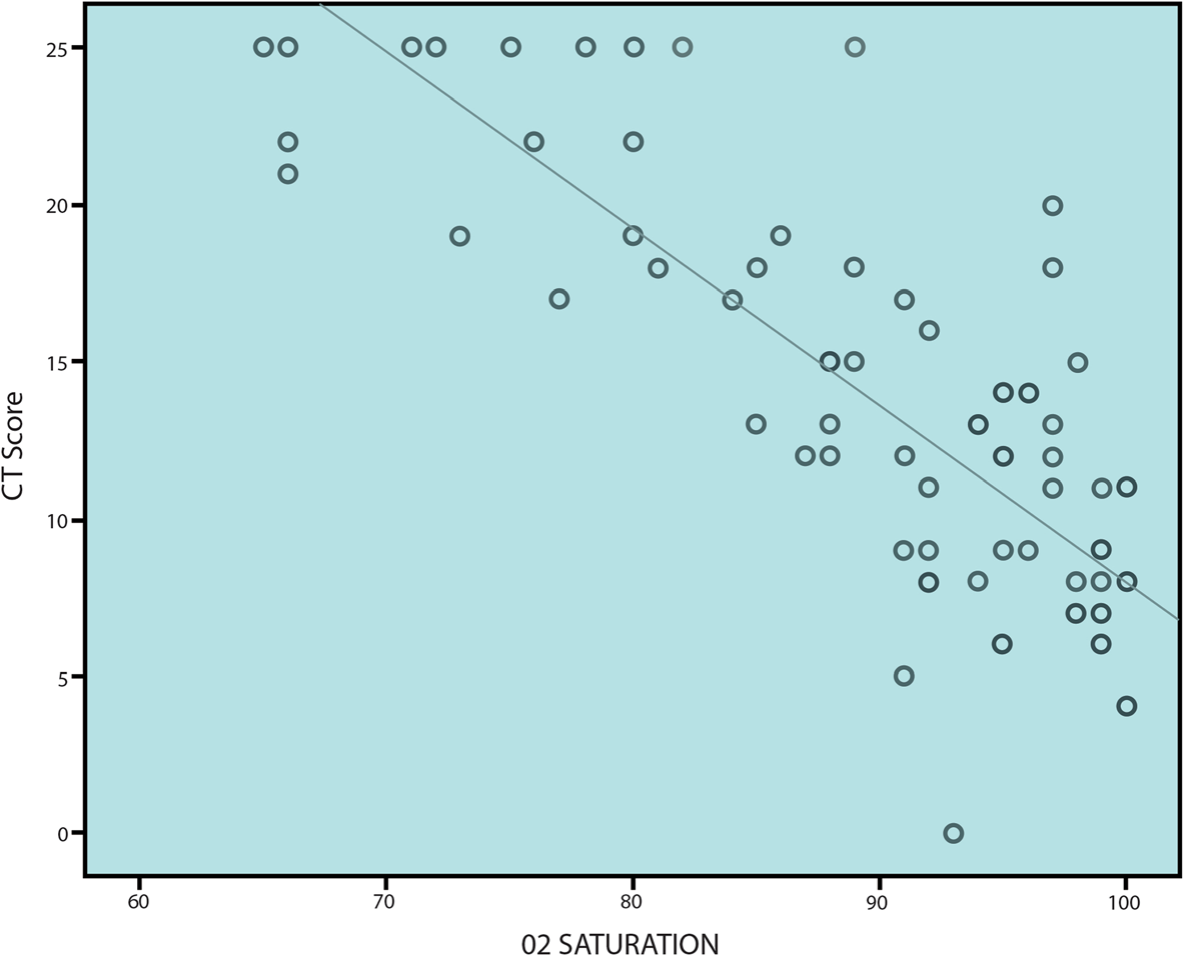
Scatter correlation graph between O_2_ saturation and CT-severity score (strong negative correlation)

**Fig. 5:**
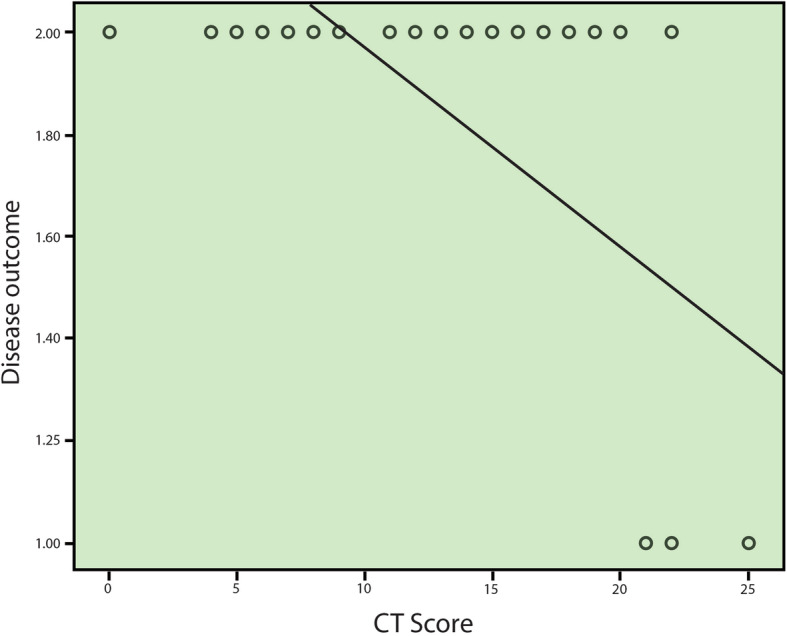
Scatter correlation graph between CT severity score and patients’ outcome (moderate negative correlation)

CT-SS ranged from 0 to 25 with mean ± SD 12.22 ± 6.02. It showed significant negative correlation with O_2_ saturation and outcome of the patients (*r* − 0.73/*p* 0.001 and *r* − 0.56/*p* 0.001 respectively) (Table 4 and Figs. 4 and 5).

**Table 4:** Correlation between CT-severity score and both O_2_ saturation and disease outcome in patients with COVID-19 infection

	CT score	
	***R***	***P*** value
**O**_**2**_ **saturation**	− 0.79	.000
**Disease outcome**	− 0.56	.000

Multivariate regression analysis of the most relevant risk factors for severity among patients with COVID-19 revealed that older age, male gender, smoking, hypertension, low O_2_ saturation, increased CT score, high serum ferritin, and high D-dimer level are the most significant risk factors (Table 5).

**Table 5:** Binary logistic regression analysis for severity among patients with COVID-19 infection

Variables	Wald	***P*** value	OR	95% CI	
				Lower	Upper
**Age**					
≥ 65 years **vs** < 65 years	11.87	0.001	4.93	2.74	9.25
**Gender**					
Male **vs** female	6.94	0.008	2.18	1.22	3.91
**Smoking history**					
Smokers **vs** non-smokers	7.83	0.005	3.31	1.43	7.63
**Hypertension**					
Yes **vs** no	5.18	0.02	2.13	1.45	6.72
**O**_**2**_ **saturation**					
≤ 93 **vs** > 93	5.76	0.01	1.98	1.13	3.46
**CT severity score**	4.40	0.03	2.75	1.06	7.11
**CBC**					
Lymphopenia **vs** normal	1.46	0.22	1.59	0.74	3.39
**S.ferritin**					
High level **vs** normal	6.48	0.006	2.13	1.22	4.75
**D.dimer**					
High level **vs** normal	10.36	0.001	3.88	1.69	8.85

The Wald test (also called the Wald Chi-squared test) is a way to find out if explanatory variables in a model are significant. “Significant” means that they add something to the model; variables that add nothing can be deleted without affecting the model in any meaningful way

Few patients (9/240) whom were confirmed by RT-PCR had extra-pulmonary manifestations as follows: two cases were diagnosed as myocarditis after normal diagnostic coronary catheterization, three cases had dural venous sinus thrombosis on brain magnetic resonance venography (MRV), two patients with acute lower limb ischemia by Doppler study and CT angiography (a case of complete occlusion of peroneal artery and another one with anterior tibial artery occlusion), and two patients with acute infarction of middle cerebral artery territory.

## Discussion

By the end of 2019, many cases with a novel febrile chest infection had been reported in Wuhan City, China. Clinical and CT imaging share characteristic findings. Our study has depended primarily on the HRCT to screen cases with clinical and epidemiological data suspicious for COVID-19 infection even with negative or unavailable RT-PCR testing. It has met the recent WHO guidelines [14].

The current study did not depend primarily on RT-PCR. This was supported by Bai et al. [15] who stated that CT chest had higher sensitivity in relation to RT-PCR, yet the obstacle was to differentiate between COVID-19 pneumonia and other viral pneumonia as well as the organizing pneumonia. Fang et al. [16] has reported 98% sensitivity for CT chest compared to 71% for RT-PCR.

Like Wu et al. [17], high fever and dyspnea as a symptom of lower respiratory tract involvement were significantly higher in patients belonged to the severe group compared to the upper respiratory tract symptoms.

On analysis of the imaging features, the bilateral multi-lobar affection with peripheral distribution was the commonest interpreting finding, and GGOs were the most encountered pattern; other reported patterns were consolidations and crazy paving as well as the septal lines. Similar outcome was stated by many studies [6, 18–23].

Thirty-six cases had mediastinal lymphadenopathy (15%), and 2 cases with pleural effusion (0.8%) have been reported; this low proportion was in concordance with Bernheim et al. [24]; also, low percentage of cases had septal lines representing (18.3%) similarly with Pan et al. [10].

In agreement with Zhao et al. [25] who concluded that old age, diffuse pulmonary affection, and more lesions’ extent as well as the presence of pleural effusion were significantly frequent among emergency cases compared to the non-emergency cases, there was no significant difference between both groups regarding the gender in their study.

Older age, male sex, smoking, low O_2_ saturation, increased CT score, and high serum ferritin and high D-dimer level are the most significant risk factors; similar outcome has been reported by Li et al. [26] and Wu et al. [17]

Hypertension as co-morbidity was an established risk factor for severity and poor patients’ outcome, similarly with Guan et al. [27], Wu et al. [17], Li et al. [26], and Zhang et al. [28]. As it is known that ACE2 is a vital regulator for cardiac functions, thus it may explain the high frequency of severe COVID-19 pneumonia among the hypertensive patients.

The current study elicited a strong negative correlation between the oxygen saturation and the CT-SS (*r* − 0.73/*p* 0.001), and this was different from the weak negative correlation reported by Wang et al. [29] (*p* < 0.05 and *r* − 0.446). There were significant correlations between the degree of pulmonary affection by COVID-19 infection and the main clinical symptoms and laboratory results, and this was in concordance with Wu et al. [30]. The current study agreed with Zhao et al. [31] who recommended clinical correlation with CT findings.

The globally disseminated COVID-19 infection outbreak is better to be aborted by the recommended protective measures.

## Limitation of the study

The shortage of RT-PCR to include all cases was the main limitation. Other issues included somehow variable duration from the onset of the symptoms and seeking medical advice and investigations. Besides no hospital-based electronic registry with self-recording of the co-morbidities.

## Conclusion

Regarding the current global pandemic of coronavirus infection, HRCT chest is the modality of choice for detecting COVID-19 pneumonia. It tells the clinicians about severity using the severity score in interpretation. Older age, male sex, smoking, hypertension, low O_2_ saturation, increased CT score, high serum ferritin, and high D-dimer level are the most significant risk factors and correlated with poor patients’ outcome and mortality.

## Recommendations

We recommend follow-up for the recovered cases to assess probable subsequent pulmonary fibrosis, also to pay attention to extra-pulmonary manifestations of COVID-19 infection, and to wait for larger scale studies to increase the awareness and knowledge about the disease and to strengthen the statistical analyses.

## Data Availability

Data will be available upon request via contacting the corresponding author.

## Abbreviations

HRCT: High-resolution computed tomography
COVID-19: Coronavirus disease 2019
SARS-CoV-2: Severe acute respiratory syndrome coronavirus 2
ACE 2: Angiotensin-converting enzyme 2
CT: Computed tomography
ICU: Intensive care unit
CT-SS: Computed tomography severity score
O_2_: Oxygen
RT-PCR: Real-time transcriptase polymerase chain reaction
MRV: Magnetic resonance venography
GGOs: Ground glass opacities
HTN: Hypertension
CRP: C-reactive protein
CBC: Complete blood count
DM: Diabetes mellitus
LFT: Liver functions tests
KFT: Kidney functions tests
